# Stigma as a Social Determinant of Health: A Policy Classification Framework and Call to Action

**DOI:** 10.1111/1468-0009.70120

**Published:** 2026-08-27

**Authors:** LISA CARTER‐BAWA

**Affiliations:** ^1^ Hackensack Meridian Health Center for Discovery and Innovation, Cancer Prevention Precision Control Institute

**Keywords:** social determinants of health, social stigma, fundamental cause theory, health policy, health equity, population health, structural stigma, policy classification framework

## Abstract

**Policy Points**

- Stigma functions as a social determinant of health (SDOH), producing population health inequities through resource deprivation, social exclusion, constrained health care, physiological stress, and diminished agency—yet it lacks formal SDOH classification.
- Because stigma operates as a fundamental cause of health inequalities, policy responses must extend beyond health care systems to encompass housing, employment, education, and social participation.
- Discrimination is already classified as an SDOH; formal stigma classification would address the broader ecosystem that enables discrimination and the anticipated, internalized, and structural forms that produce harm even in its absence.

**Context:**

Despite substantial evidence that stigma produces measurable health inequities across conditions and populations, stigma remains absent from major social determinants of health (SDOH) frameworks. This absence persists even though discrimination—one manifestation of the broader stigma ecosystem—is already classified as an SDOH.

**Methods:**

Drawing on fundamental cause theory, we derive five criteria for SDOH classification and systematically evaluate stigma against each. We synthesize evidence from systematic reviews and meta‐analyses across HIV, mental illness, lung cancer, obesity, and structural stigma research. Lung cancer serves as a running exemplar throughout.

**Findings:**

Stigma meets all five criteria for SDOH classification: structural embedding in policies and institutions, health impact through multiple pathways extending well beyond health care systems, contribution to systematic health inequities, modifiability through multilevel intervention, and measurement feasibility through validated instruments. We identify five pathways through which stigma produces population health harm: resource deprivation, social exclusion, constrained health care access and quality, physiological stress and weathering, and diminished agency and identity.

**Conclusions:**

We propose the stigma‐as‐social‐determinant (SSD) classification framework as a policy translation tool that specifies the infrastructure needed to operationalize formal recognition. Critically, because stigma operates as a fundamental cause whose health effects extend far beyond clinical encounters, policy translation must not flow primarily through health care systems. Anti‐stigma policy must engage housing, employment, education, and social policy alongside—not subordinate to—health care reform.

Social determinants of health (sdoh)—the conditions in which people are born, grow, live, work, and age—are now recognized as foundational drivers of health inequities.^1^, ^2^ Major frameworks including Healthy People 2030 and the World Health Organization's Commission on Social Determinants have codified factors such as housing instability, food insecurity, transportation barriers, social isolation, and discrimination as SDOH requiring systematic measurement and intervention.^3^, ^4^ Notably absent from these frameworks is stigma.

This absence persists despite substantial evidence that stigma operates through mechanisms consistent with recognized SDOH, produces measurable health inequities across multiple conditions, and represents a modifiable structural factor amenable to intervention.^5^, ^6^ The omission is particularly striking given that discrimination—one manifestation of the broader stigma ecosystem—is already classified as an SDOH. Recent scholarship on structural drivers of health has called for examining “the wider set of forces and systems shaping the conditions of daily life,”^7^ yet stigma has not been formally incorporated into this agenda. International scholars have similarly argued that stigma functions as a social relational determinant of health, producing inequities through mechanisms that extend well beyond individual encounters.^8^

Consider lung cancer, the leading cause of cancer death in the United States. Despite causing more deaths than breast, colorectal, and prostate cancers combined, lung cancer research has historically received disproportionately low funding relative to mortality burden. Individuals eligible for lung cancer screening frequently avoid health care settings due to anticipated judgment about their tobacco history.^9^ A systematic review found that stigma and nihilism negatively impact lung cancer outcomes across multiple domains.^10^ Health care providers may communicate, explicitly or implicitly, that a diagnosis was “brought on” by behavior.^11^ But the harm extends well beyond clinical encounters: people with lung cancer report social withdrawal, employment discrimination, strained family relationships, and a pervasive sense that their suffering is deserved—consequences that produce health harm independent of health care access or quality.^10^, ^12^ These dynamics illustrate how structural, anticipated, and enacted stigma converge to produce population health inequities through both medical and non‐medical pathways. Lung cancer serves as a paradigmatic case for understanding how stigma functions as a SDOH, and we return to this exemplar throughout.

This paper argues for formal recognition of stigma as a SDOH. Importantly, the case for stigma's SDOH status has been made before, most notably by Hatzenbuehler and colleagues^5^ who argued persuasively that stigma functions as a fundamental cause of population health inequalities. This contribution is therefore not a new theory of stigma or a first‐principles conceptualization. Rather, we offer a policy translation framework that derives classification criteria explicitly from fundamental cause theory, maps stigma onto those criteria, and outlines the policy infrastructure that would operationalize formal recognition—including, critically, policy mechanisms that extend beyond health care systems to address stigma's population‐level effects. Once classified, stigma's mechanisms could then be systematically addressed using existing SDOH implementation tools such as those proposed by Thimm‐Kaiser and colleagues.^13^

## Deriving Criteria for SDOH Classification From Fundamental Cause Theory

Before evaluating stigma's SDOH status, we must establish the criteria by which any factor warrants formal classification. We ground these criteria in fundamental cause theory (FCT), which Link and Phelan developed to explain why the association between socioeconomic status and mortality persists despite radical changes in diseases and risk factors.^14^ FCT holds that a fundamental cause influences multiple disease outcomes, affects health through multiple risk factors and mechanisms, involves access to resources that can be used to avoid risks or minimize disease consequences, and reproduces its association with health over time through the replacement of intervening mechanisms.^14^, ^15^ Hatzenbuehler and colleagues subsequently extended FCT to argue that stigma itself functions as a fundamental cause of population health inequalities.^5^

We propose that FCT provides the theoretical foundation for SDOH classification criteria. Analysis of factors already recognized as SDOH—including housing instability, food insecurity, transportation barriers, social isolation, and discrimination^3^, ^4^—reveals that each exhibits the properties FCT identifies: structural embedding that distributes resources unequally, health effects operating through multiple replaceable mechanisms, and the reproduction of inequities over time. From these theoretical and empirical foundations, we derive the following five criteria for SDOH classification.

### Criterion 1: Structural Embedding

Derived from FCT's core premise that fundamental causes are social conditions operating through the unequal distribution of resources across social structures,^7^, ^16^ this criterion requires that the factor be embedded in social, economic, or institutional structures rather than operating solely at the individual level. Housing instability, for example, reflects labor markets, zoning policies, and systemic inequities—not merely individual choices.

### Criterion 2: Health Impact Through Multiple Pathways

Directly paralleling FCT's criterion that a fundamental cause affects health through multiple risk factors and mechanisms,^14^, ^17^ SDOH classification requires that the factor produce health effects through identifiable biological, behavioral, psychological, and social mechanisms. Critically, as Lantz and colleagues have argued, these pathways must not be reduced to health care–centric mechanisms alone; population health determinants operate through pathways that extend well beyond clinical care.^18^, ^19^ Food insecurity, for example, affects health through nutritional deficiency, chronic stress, medication non‐adherence, social isolation, and diminished economic productivity—pathways that are only partially mediated by health care.

### Criterion 3: Contribution to Health Inequities

FCT predicts that fundamental causes produce patterned inequities across populations, not random variation in health outcomes.^20^ SDOH classification therefore requires that the factor differentially burden populations in ways that produce systematic health disparities. Transportation barriers disproportionately affect rural, low‐income, and disability communities, contributing to unequal access to employment, education, social participation, and health care.

### Criterion 4: Modifiability

FCT's insight that fundamental causes reproduce health inequities through the replacement of intervening mechanisms implies that interventions targeting single mechanisms will be insufficient—but the cause itself may be modifiable through structural intervention.^21^ This represents our extension of FCT into policy logic: SDOH classification requires that the factor be amenable to intervention through policy, institutional reform, or community action. Social isolation, for example, can be addressed through community programs, built environment changes, and social policy reform.

### Criterion 5: Measurement Feasibility

This criterion is our addition to the classification framework—a pragmatic requirement for policy translation that FCT, as a theoretical framework, does not address.^7^, ^22^ SDOH classification depends on the capacity for systematic surveillance, screening, and documentation. Without measurement feasibility, a factor cannot be incorporated into the administrative and clinical infrastructure through which SDOH policy operates.

Four of these five criteria map directly onto fundamental cause logic: structural embedding follows from FCT's grounding in social conditions and the unequal distribution of flexible resources (Criterion 1); health impact through multiple pathways parallels FCT's multiple‐mechanism premise (Criterion 2); contribution to inequities follows from FCT's account of how fundamental causes shape multiple disease outcomes in patterned, non‐random ways (Criterion 3); and modifiability extends FCT's explanation of how causes persist through the replacement of intervening mechanisms into policy logic (Criterion 4). The fifth criterion, measurement feasibility, has no antecedent in FCT and is our pragmatic addition for policy translation. This theoretical grounding is deliberate: rather than proposing classification criteria ad hoc, we show that the criteria for SDOH classification follow from the same theoretical framework that Hatzenbuehler and colleagues used to establish stigma as a fundamental cause. The implication is that if stigma is a fundamental cause of health inequalities—as the evidence strongly suggests—then it necessarily meets the criteria for SDOH classification derived from that same theory.

## Stigma Mapped Onto SDOH Classification Criteria

### Criterion 1: Structural Embedding

Stigma is embedded in policies, institutions, cultural norms, and media representations.^6^, ^23^ Structural stigma—defined as societal‐level conditions that constrain opportunities for stigmatized groups—manifests in funding disparities (lung cancer's research funding gap relative to mortality burden), employment discrimination (hiring bias against individuals with mental health histories), insurance exclusions (historical limitations on substance use treatment coverage), and legal frameworks (criminalization of HIV exposure).^24^, ^25^ Tyler's analysis of stigma as “machinery of inequality” demonstrates how stigma is not merely an interpersonal phenomenon but a form of structural power—reproduced through institutions, governance, and media—that shapes the distribution of resources and life chances.^26^ Hatzenbuehler and colleagues have demonstrated that structural stigma at the state level predicts health outcomes for LGBTQ+ populations independent of individual experiences.^27^ Brown and Homan have argued that such structural forms of oppression operate as fundamental drivers of population health requiring systematic measurement and policy attention.^7^

### Criterion 2: Health Impact Through Multiple Pathways

Stigma produces health harm through at least five distinct pathways, consistent with FCT's prediction that a fundamental cause affects health through multiple replaceable mechanisms.^5^, ^14^ These pathways—which we elaborate in detail below—include resource deprivation (employment, housing, education, social capital), social exclusion and diminished social participation, constrained health care access and quality, physiological stress and weathering, and diminished agency and identity.^5^, ^28^, ^29^ Critically, consistent with Lantz and colleagues' caution against medicalizing population health,^18^ the majority of these pathways operate independently of health care systems. Even when access to quality care is controlled for, members of stigmatized populations show significantly elevated morbidity and mortality,^5^, ^27^ confirming that stigma's health impact extends well beyond clinical encounters.

### Criterion 3: Contribution to Health Inequities

Stigma produces systematic health disparities across conditions and populations. Meta‐analyses demonstrate that HIV stigma predicts late presentation for care with an odds ratio of 2.4, a magnitude comparable to other recognized SDOH.^30^ Lung cancer stigma contributes to delayed diagnosis and reduced treatment intensity.^10^, ^11^ Mental illness stigma reduces help‐seeking across cultural contexts.^31^ Weight stigma produces avoidance of health care and diminished quality of care received.^32^ The discrimination literature—which captures only enacted stigma—demonstrates overwhelmingly that non‐medical pathways between discrimination and adverse health persist even when access and quality of care are controlled for.^20^, ^33^ Critically, stigma burden is not randomly distributed: it concentrates among populations already marginalized by race, class, and other social positions, compounding existing inequities.^34^

### Criterion 4: Modifiability

Stigma is amenable to intervention across multiple levels. Provider‐level interventions (contact‐based education, communication training) have demonstrated efficacy in reducing stigmatizing attitudes and behaviors.^35^, ^36^ Structural interventions (policy change, media campaigns, funding reallocation) can modify the conditions that sustain stigma.^37^ Broady and colleagues' development of stigma indicators for bloodborne viruses and sexually transmissible infections in Australia demonstrates that stigma monitoring can be integrated into national surveillance systems, enabling population‐level tracking of stigma burden and the evaluation of anti‐stigma interventions.^38^ While challenges remain in scaling stigma‐reduction interventions—a limitation we address below—the evidence base supports intervention amenability in principle.

### Criterion 5: Measurement Feasibility

Validated instruments exist for measuring stigma across conditions and domains. Cataldo's Lung Cancer Stigma Scale,^39^ the HIV Stigma Scale,^40^ and measures of mental illness stigma^41^ enable systematic assessment. The Health Stigma and Discrimination Framework provides a common measurement approach applicable across conditions.^6^ While integration into electronic health records and routine clinical workflows requires implementation research, analogous to early efforts in SDOH screening,^42^ the fundamental feasibility of stigma measurement is established.

In sum, stigma meets all five criteria for SDOH classification. The question is not whether stigma qualifies—it is why formal classification has not yet occurred.

## Existing Theoretical Frameworks: Contributions and Limitations

The past two decades have witnessed substantial theoretical development in stigma science. These frameworks have advanced our understanding of stigma's mechanisms but have not, for reasons both substantive and practical, translated into formal SDOH classification or the policy infrastructure that would follow.

Link and Phelan's seminal 2001 *Annual Review of Sociology* paper established the foundational definition of stigma as the co‐occurrence of labeling, stereotyping, separation, status loss, and discrimination in a context of power asymmetry.^43^ This conceptualization moved beyond Goffman's individual‐level focus^44^ to position stigma as a social process embedded in power structures. Building on this foundation, Hatzenbuehler and colleagues' (2013) *American Journal of Public Health* paper made the most explicit prior argument for stigma's health significance,^5^ arguing that stigma's pervasiveness, its disruption of multiple life domains, and its corrosive impact on populations warrant recognition “alongside the other major organizing concepts for research on social determinants of population health.” This paper provided the strongest prior case for stigma's SDOH status—yet it stopped short of proposing specific policy mechanisms that would operationalize such classification.

Stangl and colleagues' 2019 Health Stigma and Discrimination Framework proposed a “global, crosscutting framework” applicable across health conditions,^6^ tracing stigma from drivers and facilitators through manifestations to outcomes across multiple socio‐ecological levels. This framework enabled cross‐condition comparison and provided a common language for stigma research. More recently, Tyler's analysis of stigma as “machinery of inequality” has situated stigma within broader structures of social power and governance,^26^ while Addison and colleagues have explicitly framed stigma relations as a SDOH, demonstrating through qualitative research how stigma produces health harm through mechanisms that participants experience as integral to their social conditions.^8^

The SSD Classification Framework builds on all of these contributions. We do not reconceptualize stigma; rather, we complete the translation from academic recognition to policy classification by specifying both the criteria for SDOH classification (grounded in FCT) and the policy infrastructure that would operationalize formal recognition. Table 1 summarizes how the SSD Classification Framework relates to prior work.

**Table 1:** Relationship of the SSD Classification Framework to Prior Work

Dimension	Link & Phelan / Hatzenbuehler et al.	Stangl et al. Health Stigma Framework	SSD Classification Framework
**Primary Purpose**	Establish stigma as fundamental cause of health inequalities	Guide research and intervention development across conditions	Justify and operationalize SDOH classification; specify policy infrastructure
**Theoretical Foundation**	FCT	Socio‐ecological model; cross‐condition synthesis	FCT applied to classification logic
**Novel Contribution**	Linked stigma to population health through resource depletion	Cross‐condition applicability and measurement	SDOH classification criteria derived from FCT; policy infrastructure specification
**Orientation**	Theory and research	Research and practice	Policy and systems change
**Scope of Pathways**	Psychological and structural	Health‐related stigmas across conditions	Population health: resource deprivation, social exclusion, health care, physiological stress, constrained agency

FCT, fundamental cause theory; SDOH, social determinant of health; SSD, stigma‐as‐social‐determinant.

## The SSD Classification Framework

**Figure 1:**
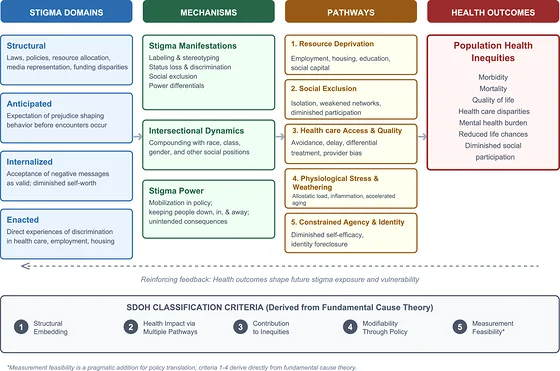
The Carter‐Bawa Stigma‐as‐Social Determinant (SSD) Classification Framework. [Colour figure can be viewed at wileyonlinelibrary.com] Four interconnected stigma domains operate through mechanisms including stigma manifestations, intersectional dynamics, and stigma power to produce population health inequities through five pathways—three of which (resource deprivation, social exclusion, and physiological stress) operate largely independent of healthcare systems. The framework demonstrates that stigma meets all five proposed criteria for SDOH classification, four derived from fundamental cause theory and one (measurement feasibility) added as a pragmatic requirement for policy translation.

The SSD Classification Framework synthesizes insights from prior conceptualizations while explicitly orienting toward policy translation. We propose four interconnected stigma domains operating through five pathways to produce population health inequities (Figure 1). Critically, we distinguish stigma from discrimination—already recognized as an SDOH—by positioning discrimination as one manifestation within the broader stigma ecosystem, and we identify pathways to health harm that extend well beyond health care systems.

### Four Stigma Domains

Drawing on established conceptualizations,^5^, ^6^, ^43^ we identify four interconnected stigma domains. (1) *Structural stigma* encompasses the societal‐level conditions, cultural norms, and institutional policies that constrain opportunities, resources, and wellbeing for stigmatized groups.^24^, ^25^ In lung cancer, structural stigma manifests in decades of federal funding disparities, media representations emphasizing personal culpability, and clinical guidelines that frame screening eligibility through behavioral history rather than individual risk assessment. (2) *Anticipated stigma* refers to the expectation of prejudice and discrimination that shapes behavior before any enacted stigma occurs.^45^ For individuals eligible for lung cancer screening, anticipated stigma produces avoidance of health care settings, social withdrawal from potentially supportive networks, and reluctance to disclose health concerns to employers or family members. (3) *Internalized stigma* involves the acceptance of negative societal messages as personally valid, producing shame, diminished self‐worth, and acceptance of adverse conditions as deserved.^46^ A person with a history of tobacco use may internalize societal blame, accepting suboptimal outcomes as appropriate. (4) *Enacted stigma* encompasses direct experiences of discrimination, prejudice, and differential treatment^6^—in health care, employment, housing, social relationships, and institutional encounters.

### Five Pathways to Population Health Inequities

A critical contribution of this framework is the identification of pathways that extend beyond health care systems. Consistent with FCT's prediction that a fundamental cause operates through multiple replaceable mechanisms,^14^ and heeding Lantz and colleagues' warning against medicalizing public health policy,^18^, ^19^ we identify five pathways through which stigma produces population health harm. Four of these five—resource deprivation, social exclusion, physiological stress and weathering, and constrained agency and identity—operate largely independent of health care systems; only the third (health care access and quality) is primarily health care–mediated.

*Pathway 1: Resource Deprivation*. Stigma depletes the flexible resources that FCT identifies as central to health advantage—money, knowledge, power, prestige, and beneficial social connections.^5^, ^14^ People who experience stigma face employment discrimination, housing instability, educational barriers, and erosion of social capital. These resource losses produce health harm independent of health care access. A person with a mental health history denied employment experiences downstream health effects—poverty, food insecurity, chronic stress—regardless of whether they have access to quality mental health care. In lung cancer, structural stigma has produced decades of disproportionately low research funding, reducing the therapeutic options available to an entire patient population—a resource deprivation operating at the population level that no amount of individual clinical care can remedy. Elraz and Remnant's research on stigma in mental health work settings demonstrates how employment‐based stigma constrains economic resources and social participation for individuals working against the backdrop of extreme marginalization.^47^

*Pathway 2: Social Exclusion and Diminished Participation*. Stigma produces social isolation, weakened social networks, reduced civic engagement, and exclusion from community life. The evidence on social isolation as a health risk factor is robust, with effect sizes comparable to established risk factors including smoking.^48^ Stigma‐driven social exclusion operates through both withdrawal (the stigmatized person retreats from social engagement) and rejection (others distance themselves from the stigmatized individual). For people with lung cancer, social withdrawal may sever connections to networks that could provide emotional support, health information, or practical assistance. Tyler's analysis demonstrates how stigma functions as a mechanism of social sorting—keeping people down, keeping people in, and keeping people away—that constrains social participation in ways that produce health harm regardless of health care availability.^26^

*Pathway 3: Health Care Access and Quality*. Anticipated and enacted stigma affect health care utilization and quality. People avoid or delay care, screening, and treatment due to anticipated judgment;^28^, ^49^ when they do engage, enacted stigma produces differential treatment—less time, less empathy, less aggressive intervention.^32^, ^50^ Meta‐analyses across conditions consistently demonstrate that stigma predicts later presentation, reduced engagement, and premature discontinuation of care.^30^, ^31^ This pathway remains important, but it is now situated as one of five rather than the dominant framing. Addressing health care stigma alone will not resolve stigma's population health impact—a point underscored by the persistence of health inequities among stigmatized populations even when health care access is equalized.^5^, ^27^

*Pathway 4: Physiological Stress and Weathering*. Chronic exposure to stigma activates stress response systems, producing allostatic load, inflammation, and accelerated biological aging—what Geronimus has termed “weathering.”^51^ These physiological effects operate independently of health care access and may operate independently of conscious psychological distress. A person may not consciously experience stigma‐related distress and still show elevated cortisol, inflammatory markers, and cardiovascular risk. Hatzenbuehler and colleagues have documented the biological embedding of structural stigma, demonstrating that state‐level stigma predicts physiological stress markers among LGBTQ+ populations.^27^ For people with lung cancer, the chronic stress of carrying a stigmatized diagnosis compounds the biological burden of disease itself.

*Pathway 5: Constrained Agency and Identity*. Internalized stigma reshapes how people understand themselves and what they believe they deserve. This pathway extends beyond psychological distress to encompass identity foreclosure, diminished self‐efficacy, acceptance of suboptimal conditions, and reduced engagement in health‐protective behaviors.^29^, ^46^ Critically, this pathway operates even in the absence of enacted discrimination: a person who has internalized societal messages may never seek help not because of anticipated judgment, but because they have accepted that they are undeserving. For people with lung cancer, internalized stigma may lead not only to avoiding screening but to accepting a late‐stage diagnosis as deserved, declining aggressive treatment, or failing to advocate for oneself in clinical or social encounters.

### Distinguishing Stigma from Discrimination

A crucial conceptual move distinguishes the SSD Classification Framework. Discrimination—differential treatment based on group membership—is already recognized as an SDOH.^3^, ^20^ We argue that discrimination represents only one manifestation of the broader stigma ecosystem. Discrimination is enacted stigma. But anticipated stigma produces harm before any discriminatory act occurs. Internalized stigma produces harm even in the absence of discrimination. And structural stigma shapes the conditions that enable discrimination while extending beyond discrete discriminatory acts.^5^, ^23^ The discrimination literature itself demonstrates overwhelmingly that the non‐medical pathways between discrimination and adverse health persist even when access and quality of care are controlled for^20^, ^33^—evidence that underscores the need to look beyond health care–mediated mechanisms. Recognizing discrimination as an SDOH while ignoring the broader stigma ecosystem addresses symptoms while missing underlying causes.

### Stigma Power, Weaponization, and Unintended Policy Consequences

Any framework proposing stigma as an SDOH must reckon with a fundamental paradox: stigma is not merely an unintended byproduct of social processes but a form of power that can be deliberately mobilized—sometimes with harmful consequences, including by well‐intentioned public health campaigns.^26^, ^52^ Link and Phelan identified three functions of stigma: keeping people down (exploitation and domination), keeping people in (social control and norm enforcement), and keeping people away (avoidance of perceived threat).^52^ Tyler's analysis extends this to show how stigma operates as “machinery” embedded in governance and institutions—not merely attitudes held by individuals.^26^

The lung cancer exemplar illustrates this paradox acutely. Anti‐tobacco public health campaigns—among the most successful behavior‐change interventions in history—succeeded in part by stigmatizing tobacco use. Smoking rates declined substantially. But these campaigns simultaneously created a stigma environment so powerful that it now impedes the very people they aimed to protect from accepting life‐saving screening. The weaponization of stigma for one policy goal (reducing tobacco use) has produced collateral harm for another (early detection of lung cancer). This is not a unique case: stigma has been mobilized in drug policy, immigration enforcement, and mental health commitment proceedings with consequences that extend far beyond their intended scope.^26^

The implication for SDOH classification is that anti‐stigma policy cannot be reduced to simply “reducing stigma.” Policy must attend to stigma's functions as a form of social power—who wields it, to what ends, and with what unintended consequences. SDOH classification should prompt not only measurement and intervention, but critical analysis of how stigma is produced, sustained, and deployed within policy systems themselves.

### Stigma, Racism, and Intersecting Determinants

A question arises: Is stigma a crosscutting modifier of other SDOH, or a standalone determinant? We propose that stigma functions as both. Stigma operates as a standalone SDOH when condition‐based stigma (e.g., lung cancer, mental illness, HIV) produces health harm independent of other social positions. Simultaneously, stigma intersects with and amplifies other determinants: racialized stigma compounds condition‐based stigma, such that a Black person with a history of tobacco use may face both racial discrimination and condition‐based stigma in health care encounters, employment settings, and social relationships.^34^, ^53^

This intersectional reality has implications for both measurement and intervention. Assessment instruments must capture both condition‐specific stigma and its intersection with racial, gender, and class‐based discrimination.^54^ Interventions must address both condition‐specific stigma mechanisms and the broader structures of racism and marginalization that shape stigma's distribution and impact.^7^, ^33^ Brown and Homan's call for research examining how overlapping forms of structural oppression jointly affect health distribution is directly applicable here.^7^

## Evidence Supporting Stigma as an SDOH

The evidence base supporting stigma's health consequences is substantial, spanning systematic reviews, meta‐analyses, and intervention trials across multiple conditions and international contexts. In HIV/AIDS, Rueda and colleagues' meta‐analysis of 64 studies demonstrated consistent associations between HIV stigma and depression, reduced social support, and lower treatment adherence,^29^ while Gesesew et al. found that stigma predicts late presentation for care with an odds ratio of 2.4.^30^ In mental illness, Corrigan and colleagues' comprehensive reviews documented stigma's effects on help‐seeking, treatment engagement, and recovery,^35^ and Clement et al.'s synthesis of 144 studies established stigma as a consistent barrier to mental health service utilization across cultural contexts.^31^ In obesity, Phelan et al.'s systematic review documented weight stigma's impact on care quality and health outcomes, including evidence of provider bias affecting clinical decision‐making.^32^ Hatzenbuehler and colleagues' 2024 *Lancet Public Health* narrative review of 98 studies established that structural stigma embedded in policies and institutions produces measurable health harm for LGBTQ+ populations.^27^

In lung cancer, Chambers et al.'s systematic review established associations between stigma, psychological distress, diminished quality of life, and reduced help‐seeking.^10^ Hamann and colleagues documented both patient‐reported stigma experiences and provider attitudes contributing to therapeutic nihilism.^11^ Cataldo's validated measures^39^ and Carter‐Harris's research demonstrating that lung cancer stigma predicts timing of medical help‐seeking behavior^55^ have enabled systematic assessment and demonstrated how stigma shapes clinical encounters. The National Lung Cancer Roundtable's Stigma & Nihilism Task Group has developed tools for stigma‐informed communication and is advancing efforts to translate this evidence into clinical practice.

Importantly, this evidence base extends beyond the United States. Addison and colleagues' qualitative research in the United Kingdom explicitly frames stigma as a SDOH, documenting how participants experience stigma as inseparable from the social conditions that shape their health.^8^ Broady and colleagues' development of national stigma indicators in Australia demonstrates the feasibility of population‐level stigma surveillance.^38^ The global scope of this evidence strengthens the case for stigma's SDOH classification while underscoring that implementation mechanisms must be adapted to diverse national and regional health systems.

## Limitations and Boundary Conditions

We acknowledge important limitations. First, stigma salience varies across conditions, populations, and contexts.^6^, ^56^ HIV stigma operates differently in communities with high versus low prevalence; lung cancer stigma may be more pronounced in regions with strong anti‐tobacco cultures. SDOH classification should not imply that all stigma operates identically; rather, it should prompt context‐specific assessment and intervention.

Second, intervention scalability remains an open question. While stigma‐reduction interventions have demonstrated efficacy in controlled trials,^35^, ^36^ evidence of scalable, system‐level implementation is limited. This represents a research gap rather than a barrier to classification—housing instability was classified as an SDOH before comprehensive solutions existed—but it should inform expectations about implementation timelines.

Third, the **r**isk of medicalizing stigma is real. Several of the policy mechanisms we propose—International Classification of Diseases (ICD) codes, clinical screening, reimbursement structures—reflect health care infrastructure and could inadvertently channel anti‐stigma work through the very clinical systems that Lantz and colleagues have shown are insufficient for addressing population health determinants.^18^, ^19^ We have sought to mitigate this risk by identifying pathways to health harm that extend well beyond health care, by calling for policy responses across housing, employment, education, and social policy sectors, and by emphasizing that health care–based interventions must complement rather than substitute for structural reform. Nevertheless, this tension warrants ongoing vigilance.

Fourth, the policy mechanisms we propose reflect primarily US health care and policy infrastructure. While the conceptual case for stigma as an SDOH applies globally, implementation mechanisms will require adaptation to national and regional health systems. The World Health Organization's SDOH framework provides a foundation for international translation,^3^ and we encourage parallel policy development across contexts.

Fifth, distinguishing stigma from related constructs remains important. Policymakers may reasonably ask how stigma differs from other psychosocial stressors competing for SDOH designation. We propose that stigma's distinguishing features—as established by FCT—include its structural embedding, its operation through marked identity and power asymmetry, its deployment of flexible resource depletion, and its reproduction of inequities through the replacement of intervening mechanisms.^5^, ^7^, ^14^

## Implications of SDOH Classification

What would change if stigma were formally recognized as a social determinant of health? We organize implications across four domains, emphasizing that—consistent with our de‐medicalized pathway model—policy responses must extend well beyond health care systems.

### Social and Economic Policy

Because stigma depletes flexible resources and constrains social participation, SDOH classification should catalyze anti‐stigma provisions in housing policy (fair housing protections for stigmatized conditions), employment policy (anti‐discrimination and reasonable accommodation frameworks), educational policy (anti‐stigma curricula and institutional climate assessment), and social policy (funding for community‐based anti‐stigma programs). These upstream interventions address stigma's structural roots rather than its downstream health care consequences.

### Health Care Practice

Within health care, SDOH classification would normalize routine stigma assessment alongside existing SDOH screening,^42^ catalyze development of stigma‐informed care protocols analogous to trauma‐informed care,^57^ and create reimbursement pathways for stigma‐reduction interventions currently limited to research contexts. However, consistent with Lantz and colleagues' caution,^18^ these health care–based reforms must be understood as necessary but insufficient—one component of a broader policy response.

### Surveillance and Measurement

Development of ICD codes for stigma‐related harm would enable documentation and tracking. Inclusion of stigma‐related measures in quality frameworks would incentivize health systems to address stigma burden. Integration into population health surveillance (e.g., Behavioral Risk Factor Surveillance System, National Health Interview Survey) would enable tracking of burden, trends, and disparities at the population level.^7^, ^22^ Broady and colleagues' Australian stigma indicator work provides a model for national implementation.^38^

### Research

SDOH classification would accelerate measurement standardization,^6^, ^22^ stimulate intervention science examining both health care and non‐health care interventions, and create demand for implementation research examining stigma's integration into routine care and social policy. Research must attend to both the effectiveness and the unintended consequences of anti‐stigma interventions, given the paradox of stigma power described above.

## Conclusion

The available evidence meets commonly accepted thresholds for SDOH classification: stigma is structurally embedded, operates through multiple pathways extending well beyond health care systems, produces systematic health inequities, is amenable to intervention, and can be measured with validated instruments. The conceptual foundations have been laid by decades of scholarship,^5^, ^6^, ^43^ and the criteria for classification follow directly from the FCT that established stigma's health significance.^14^ What remains is the translation from academic recognition to policy classification.

We call on the following actions:

1. Major SDOH frameworks (Healthy People, World Health Organization, Agency for Healthcare Research and Quality) should formally incorporate stigma as a social determinant of health.
2. Policy responses to stigma must extend beyond health care systems to encompass housing, employment, education, and social policy.
3. ICD coding committees should develop diagnostic codes for stigma‐related harm, while recognizing that coding alone is insufficient without complementary social policy.
4. Quality organizations should develop metrics for stigma assessment and stigma‐informed care.
5. Surveillance systems should incorporate validated stigma measures, building on international models such as Broady and colleagues' stigma indicator framework.^38^
6. Payers should develop reimbursement mechanisms for stigma assessment and intervention across health care and community‐based settings.

The human cost of stigma is substantial. The infrastructure to address it—across health care systems and beyond—is within reach. What is needed now is the conceptual clarity and policy will to name stigma for what it is: a SDOH deserving the same systematic, multi‐sectoral attention we now give to housing, food, and transportation. As a fundamental cause of population health inequalities, stigma cannot be resolved through health care alone. The time for stigma's formal recognition as a SDOH has arrived.

## Funding/Support

None

## Conflict of Interest Statement

None
